# Detection and Relative Quantification of Neodymium in Sillai Patti Carbonatite Using Decision Tree Classification of the Hyperspectral Data

**DOI:** 10.3390/s22197537

**Published:** 2022-10-05

**Authors:** Muhammad Qasim, Shuhab D. Khan

**Affiliations:** 1 Department of Earth and Atmospheric Sciences, University of Houston, Houston, TX 77004, USA; 2 Geoscience Advanced Research Laboratories, Geological Survey of Pakistan, Islamabad 45500, Pakistan

**Keywords:** neodymium, Rare Earth Elements, decision tree classification, hyperspectral imaging, carbonatite, Sillai Patti, neodymium index

## Abstract

A recent increase in the importance of Rare Earth Elements (REEs), proportional to advancements in modern technology, green energy, and defense, has urged researchers to look for more sophisticated and efficient exploration methods for their host rocks, such as carbonatites. Hyperspectral remote sensing has long been recognized as having great potential to identify the REEs based on their sharp and distinctive absorption features in the visible near-infrared (VNIR) and shortwave infrared (SWIR) electromagnetic spectral profiles. For instance, neodymium (Nd), one of the most abundant Light Rare Earth Elements (LREEs), has among the most distinctive absorption features of REEs in the VNIR part of the electromagnetic spectrum. Centered at ~580, ~745, ~810, and ~870 nm in the VNIR, the positions of these absorption features have been proved to be independent of the mineralogy that hosts Nd, and the features can be observed in samples as low in Nd as 1000 ppm. In this study, a neodymium index (NI) is proposed based on the 810 nm absorption feature and tested on the hyperspectral images of the Sillai Patai carbonatite samples to identify Nd pixels and to decipher the relative concentration of Nd in the samples based on the depth of the absorption feature. A preliminary spectral study of the carbonatite samples was carried out using a spectroradiometer to determine the presence of Nd in the samples. Only two of the absorption features of Nd, centered at ~745 and ~810 nm, are prominent in the Nd-rich samples. The other absorption features are either weak or suppressed by the featureless spectra of the associated minerals. Similar absorption features are found in the VNIR and SWIR images of the rock samples captured by the laboratory-based hyperspectral cameras that are processed through Minimum Noise Fraction (MNF) and Fast Fourier Transform (FFT) to filter the signal and noise from the reflectance data. An RGB false-color composite of continuum-removed VNIR reflectance bands covering wavelengths of 587.5, 747.91, and 810.25 nm efficiently displayed the spatial distribution of Nd-rich hotspots in the hyperspectral image. The depth of the 810 nm absorption feature, which corresponds to the concentration of Nd in a pixel, is comparatively greater in these zones and is quantified using the proposed NI such that the deeper the absorption feature, the higher the NI. To quantify the Nd-rich pixels in the continuum-removed VNIR images, different threshold values of NI are introduced into a decision tree classifier which generates the number of pixels in each class. The strength of the proposed NI coupled with the decision tree classifier is further supported by the accuracy assessment of the classified images generating the Kappa coefficient of 0.82. Comparing the results of the remote sensing data obtained in this study with some of the previously published studies suggests that the Sillai Patti carbonatite is rich in Nd and associated REEs, with some parts of the samples as high in Nd concentration as 1000 ppm.

## 1. Introduction

Rare Earth Elements (REEs), i.e., yttrium (Y), scandium (Sc), and the lanthanides from lanthanum (La) to lutetium (Lu), have become imperative to many modern technologies ranging from smartphones and televisions to LED light bulbs and wind turbines, and the production of green energy, electric vehicles, national defense, and more [1,2,3,4,5]. The occurrence of these metals in the Earth’s crust, their mineralogy, and different types of deposits on land and oceans are a vital part of the mineral exploration industry. So far, many studies have been conducted on the association and exploration of the REE-rich rocks and minerals to improve the understanding of the location, type, and style of REE mineralization [6,7,8,9,10,11,12,13,14,15,16]. However, the minerals rich in REEs are commonly associated with the uncommon varieties of igneous rocks, such as carbonatites and alkaline rocks, or in residual deposits formed from physical and chemical weathering of igneous rocks, pegmatites, iron-oxide, copper-gold deposits, and marine phosphates. Because of their exotic and often cryptic ore and alteration mineralogy, the exploration and mining of REEs deposits are often challenging, especially when using conventional exploration methods.

Remote sensing of the Earth’s surface is often useful in extracting geological and mineralogical information that the classical methods of surface observation may not easily acquire, e.g., dikes in the ultramafic rocks of Muslim Bagh ophiolites [17] and gneiss in the Chocolate Mountains of California [18]. It is well established by the earliest workers [19,20,21,22,23], who integrated the concept of spectroscopy and mineralogy that rocks and minerals produce some diagnostic absorption features at specific wavelengths on the electromagnetic spectrum due to multiple reasons. For instance, the absorption features in the spectral profile of minerals hosting REEs and other transition elements in the visible-near infrared (VNIR) part of the spectrum can be physically and chemically explained by electronic field transitions. Transition metals such as iron (Fe) or neodymium (Nd) can transfer the charge between their energy levels and shift between different oxidation states: ferrous (Fe^+2^) and ferric (Fe^+3^) in the case of Fe and Nd^+2^, Nd^+3^ and Nd^+4^ in case of Nd. The prominent absorption features of the trivalent neodymium, one of the most abundant Light Rare Earth Element (LREE) metals, in the VNIR part of the spectrum are roughly at ~580, ~740, ~800, and ~870 nm [24,25]. The use of these absorption features in the spectral profiles of remotely sensed data is a powerful tool for identifying rock mineralogy. The VNIR region is helpful in mapping gossans and regolith also, which may host certain REEs [26,27]. Similarly, the shortwave infrared (SWIR) region of the electromagnetic spectrum carries absorption features of clays and carbonates associated with the carbonatites and the hydrothermal alteration zones. Thus, there exists significant potential for hyperspectral remote sensing to explore the REE ore deposits.

**Figure 1:**
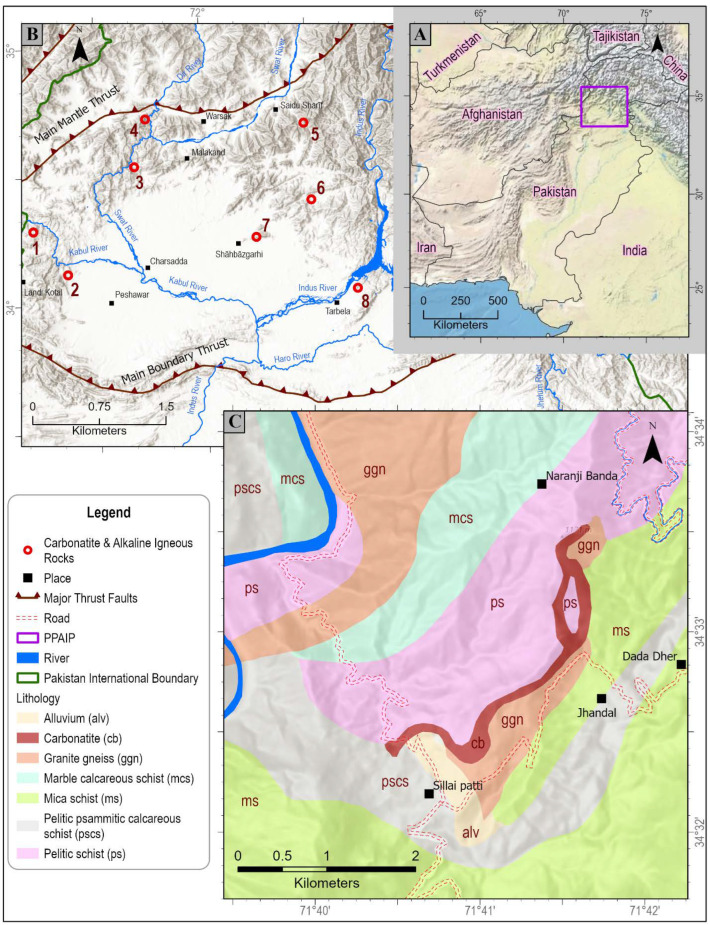
(**A**) Map showing the regional location of the Peshawar Plain Alkaline Igneous Province (PPAIP); (**B**) Carbonatites and alkaline igneous rocks of PPAIP 1. Loe-Shilman, 2. Warsak, 3. Sillai Patti, 4. Jambil, 5. Jawar, 6. Koga, 7. Shewa-Shahbazgarhi, 8. Terbela; (**C**) Geological map showing the Sillai Patti carbonatite and the surrounding rocks.

Carbonatites are mantle-derived igneous rocks that contain more than 50 modal percent of carbonates and less than 20 wt.% SiO_2_ [28,29,30,31,32]. Sillai Patti carbonatite is the second-largest carbonatite complex in Pakistan [33], after Loe-Shilman carbonatite. Therefore, a large body of data exists on the isotope dating, tectonics, and emplacement of the carbonatite, as well as the other known carbonatites and alkaline igneous rocks of Pakistan e.g., Loe-Shilman, Warsak, Jambil, Jawar, Koga, Shewa-Shahbazgarhi, and Terbela (Figure 1) [34,35,36,37,38,39]. Fewer authors have explored their REE potential [40] and none, to the authors’ knowledge, have analyzed the REE potential using remote sensing techniques. The depth and the area of the diagnostic absorption features of Nd and other REEs are dependent purely on the absolute concentration of REE atoms in rock and not the rock mineralogy [21,41]. These absorption features are produced as a result of the intraconfigurational transitions of 4f-shell electrons that are well shielded by the outer 5s and 5p shells and thus are not participating in the atomic bonding. Therefore, the absorption features are proportional to only the elemental concentration of the REEs and not the atomic vibrational phenomenon. 

This unique property makes the REEs more interesting when it comes to studying the carbonatites and other REE-hosting rocks using remote sensing techniques.

In this study, the REEs potential in the Sillai Patti carbonatite samples is analyzed in the laboratory using the spectral signatures obtained through an ASD spectroradiometer and VNIR and SWIR hyperspectral cameras. To detect the presence of Nd in the carbonatite samples, the diagnostic absorption feature of Nd at ~810 nm is targeted in the processed hyperspectral images of the samples. Based on this absorption feature, a neodymium index (NI) is defined and tested on the processed VNIR image through a machine learning algorithm, the decision tree classification. Decision tree classification is a type of supervised machine learning algorithm that can make decisions based on pre-defined classification rules. These rules are introduced at each node of a tree that is used by the algorithm to split the input data into two classes. NI threshold values in this study are used as the rules to classify the processed VNIR images of the carbonatite samples into different classes. It has been observed that the NI captured all the Nd pixels very well in the VNIR hyperspectral images and delineated the relative concentration of Nd in the carbonatite samples with a kappa coefficient of 0.817. The study shows that the hyperspectral remote sensing technique is powerful enough to identify the materials in a rock that are in parts per million (ppm). Identifying and quantifying REEs through hyperspectral remote sensing is a relatively quick, inexpensive, and less energy-consuming technique compared to instrument-based geochemical analysis. The integration of NI with the machine learning technique has been proved to be a simple, quick, and effective method of mapping the Nd concentration in the carbonatite samples by breaking the rock image into different classes based on the strength of the targeted absorption feature of Nd.

## 2. Geological Setting of the Study Area

Modern carbonatites include an active volcano, Oldoinyo Lengai, in Tanzania [42,43], a lately extinct “Early Quaternary” (<5 Ma) volcano, Khanneshin, in Afghanistan [44,45,46,47,48,49,50], and shallow, sub-volcanic bodies such as the 18 million-year-old Kaiserstuhl in the Rhine graben of Germany [51] and 20 Ma Mt Elgon in Uganda [52]. These relatively modern bodies, being volcanoes, are approximately circular in map shape and close to cylindrical or carrot-shaped up to 1–3 km in depth. Older undeformed carbonatites, such as the ca. 550 Ma Fen in Norway [53,54] and ca.550 Ma Alno in Sweden [55,56], are similarly sub-volcanic and roughly circular in outcrop. Other ancient carbonatites are structurally complex and, either locally or pervasively, gneissic. Lenticular bodies are common among them. This is considered to be a consequence of deformation [35,57]. Sillai Patti is an example of a lenticular body located in the Peshawar Plain Alkaline Igneous Province (PPAIP; Figure 1A).

PPAIP contains carbonatites, nepheline syenites, and related granites and basalts that have yielded Late Carboniferous to Permian as well as Cenozoic ages [37,58,59,60,61,62]. These ages indicate two episodes of intrusion [34,60,61] or Permian-aged intrusion followed by Cenozoic metamorphism [62,63,64,65]. Known carbonatites in PPAIP include Loe-Shilman, Jawar, Sillai Patti, and Jambil (Figure 1B). According to Le Bas et al. [60] and Tilton [34], the carbonatites of Pakistan were emplaced along the thrust faults during the Carboniferous. However, using the fission track age of apatite, Khattak et al. [33,58] suggested the Oligocene age for the emplacement of carbonatites of PPAIP. Published work and our reconnaissance observations of the PPAIP and neighboring areas have shown that all the carbonatites of the region are somewhat deformed and outcrop close to deformed slivers of ophiolite and the Main Mantle Thrust (MMT). Thus, the younger ages may be metamorphic due to the Cenozoic Himalayan collision and subsequent deformation. Except for Gohati volcanism, no other igneous activity in Oligocene in this area is reported [35].

Sillai Patti carbonatite, which outcrops about 70 km north of Peshawar, was first reported by Ashraf and Chaudhry [66]. The carbonatite body is 2–20 m wide and 12 km long and has a thrusted contact with a Paleozoic gneiss and metasediments (Figure 1C).

## 3. Methodology

**Figure 2:**
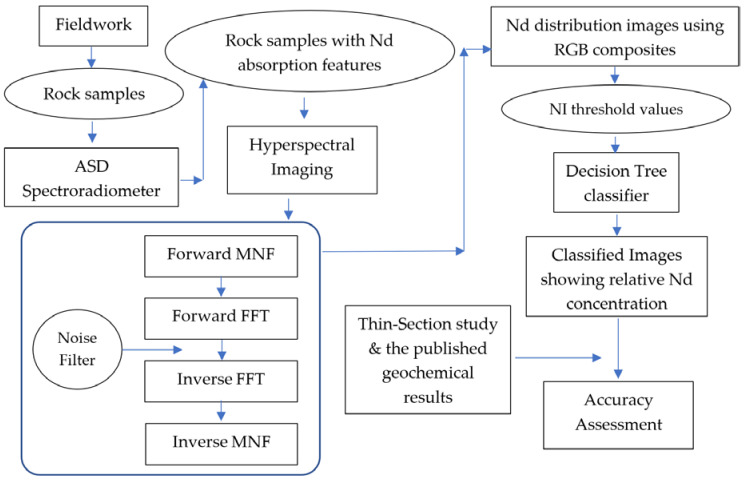
Flowchart showing the methodology adopted in the study (ASD: Analytical Spectral Device; FFT: Fast Fourier Transform; MNF: Minimum Noise Fraction; NI: neodymium index).

The approach adopted in this study is summarized as a flowchart in Figure 2.

**Figure 3:**
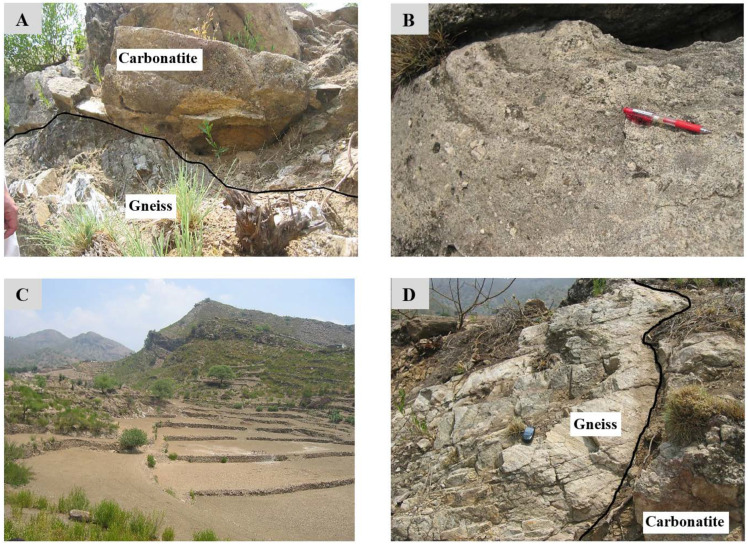
(**A**) Sillai Patti carbonatite (above) in faulted contact with gneiss of probable 510 Ma age; (**B**) typical exposure of the Sillai Patti carbonatite, amphibole is dark-colored and altered while the carbonate are light-colored; (**C**) carbonatite outcrop from a distance; (**D**) carbonatite in contact with gneiss.

**Figure 4:**
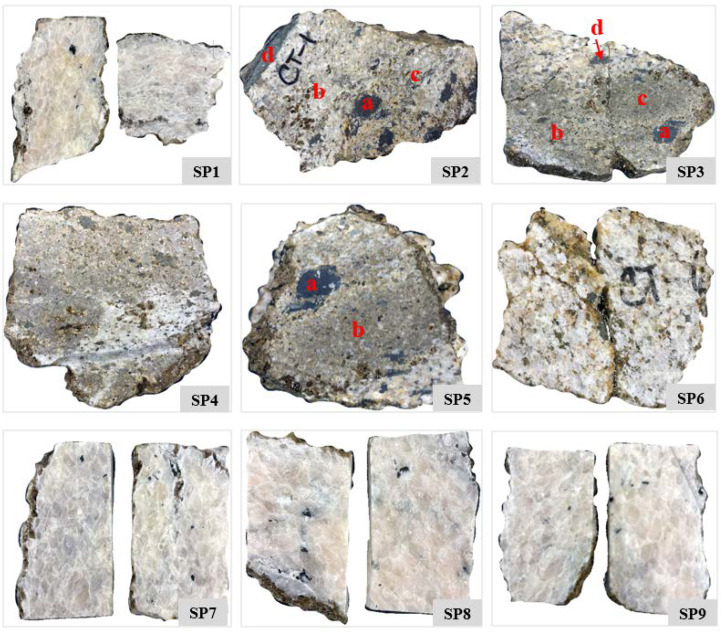
The Sillai Patti carbonatite samples collected from the field. The red-colored annotations on the samples SP2, SP3, and SP5 represent the points of the ASD spectroradiometer measurements for the spectral signatures. The spectra obtained at each of these points are shown in Figure 7.

Fieldwork was conducted in the Sillai Patti area, and nine rock samples were randomly collected along the carbonatite body (Figure 3 and Figure 4). Spectral reflectance data and laboratory-based hyperspectral images were obtained for these nine samples. Thin sections were investigated using a petrographic microscope to correlate the spectral results with the mineralogy of the samples.

### 3.1. Point Spectral Data

Rock samples were analyzed for their spectral signature in the VNIR and SWIR electromagnetic spectra using an ASD FieldSpec Pro FR spectroradiometer (Malvern Panalytical, Malvern, UK). In a controlled laboratory environment, this instrument can record the unique spectral behavior of the rocks in the form of spectral signatures within VNIR and SWIR parts of the electromagnetic spectrum ranging from 0.35 to 2.5 μm with a sampling interval of 2 to 3 μm. After instrument calibration using Spectralan, a high-intensity contact probe equipped with a halogen bulb is placed on the flat surface of the individual samples. This probe has light detectors that record the light reflecting from the samples in unique spectral signatures. Five spectra were collected for each of the nine rock samples, which were then averaged to get a single spectral profile for each sample.

### 3.2. Hyperspectral Imaging

Rock samples were imaged in the laboratory setting using SPECIM VNIR and SWIR hyperspectral cameras to record the spectral signatures between 400 and 2500 nm wavelengths. The lens used with the VNIR camera has a field of view (FOV) of 28.9° and a pixel size of 0.3 mm. With the SWIR camera, both a wide-angle lens, OLES 22.5, with an FOV of 24° and a pixel size of 0.4 mm, and a narrow-angle lens, OLES 56, with an FOV of 10° and a pixel size of ~32 μm, were used. Cameras were fixed on a stand while the samples were placed on a stage that can be moved at a constant and controlled speed to scan the samples through the camera in an along-track direction. Four halogen light bulbs of 50-watts were used to illuminate the samples. Square pixels of the scanned image were achieved by keeping a frame rate of 10 Hz. To evaluate and remove the background noises of the cameras from the image signals, dark current images were also collected by covering the lenses with their caps. Spectralon, a white reference panel, was placed next to the samples on the stage.

### 3.3. Hyperspectral Image Processing

The raw hyperspectral data were calibrated using the following pre-processing steps. Dark frames are subtracted from the collected image pixels. The raw data are then converted to at-sensor reflectance through the empirical line calibration using the Spectralon white reference recorded in the image. Next, the image artifacts and the noise usually caused by an along-track sensor are corrected. To remove this noise, which is more pronounced in the VNIR data compared to the SWIR data, the Minimum Noise Fraction (MNF) transform is used. In the first step, forward MNF rotation is performed to separate the signal and the noise in the data. Only the coherent bands with higher eigenvalues are used to transform the data from the spectral domain to the spatial domain through Forward Fast Fourier Transform (FFT). A filter is designed to highlight the noisy spatial frequency components in the forward FFT bands. The data is brought back from the spatial domain to the spectral domain through an inverse FFT which removes the noisy spatial frequency components from the images. Finally, the data is brought back to the original data space through inverse MNF, resuming the original number of spectral bands but with maximum noise removed from the data.

After processing the VNIR and the SWIR raw images, different RGB combinations of the forward MNF bands are tried to see the spatial distribution of various mineral components in the rock samples. The Pure Pixel Index (PPI) is calculated using the forward MNF bands to extract the pure pixels that contain the majority of only one of the mineral components in a pixel. These pure pixels and MNF band combinations are used to identify the pixels containing Nd. The Nd spectra are identified by comparing the pixel spectra with the reference spectra of USGS spectral libraries [67] available in the ENVI software v.5.6.

To compare the individual absorption features from a common baseline, the continuum removal is performed on the noise-removed image which normalizes the reflectance spectra using a convex hull fit over the top of the spectrum. This makes the spectra and continuum equal to 1.0 and keeps the absorption features less than 1.0. On comparing the continuum-removed VNIR reflectance spectrum of Nd extracted from different pixels of the image, it is observed that only one among all the Nd absorption features, centered on the 810 nm wavelength, is the most reliable diagnostic feature because it stays prominent even in those pixels where Nd has a low concentration and/or the spectral signature of other minerals in the pixel suppress some of the Nd absorption features. The following band ratio is designed for the neodymium index (NI) that defines the strength (depth and area) of the 810.25 nm absorption feature of Nd:

Neodymium Index (NI) = [(Band 135 + Band 153)/Band 145]

**Figure 5:**
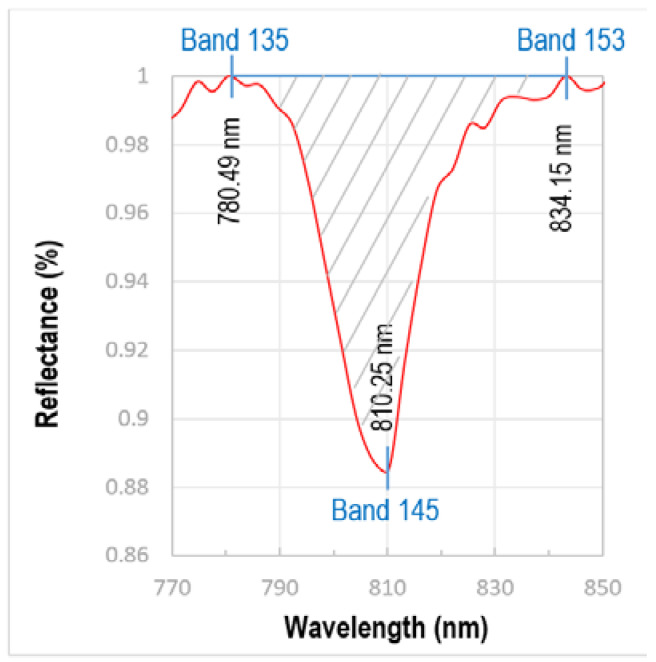
The area (the shaded part with oblique grey lines) of the absorption feature at wavelength 810.25 nm corresponds to the Nd concentration. The wavelengths 780.49 nm (band 135) and 834.15 nm (band 145) are taken as the shoulders of the Nd absorption feature.

Both the bands of the numerator, band 135 centered at 780.49 nm and band 153 centered at 834.15 nm, define the left and right shoulders of the absorption feature, whereas band 145, centered at 810.25 nm, defines the deepest point in the absorption feature (Figure 5). The higher the value of this NI, the deeper the absorption feature. As discussed earlier, the Nd absorption feature is solely the result of electron transition in the crystal lattice and is independent of the mineralogy. Therefore, a higher value of the NI is proportional to the Nd concentration in a particular pixel.

### 3.4. Hyperspectral Image Classification

To identify the Nd pixels in the hyperspectral images and to classify the images based on the relative concentration of Nd, a decision tree classifier was designed in ENVI. A decision tree classifier breaks down a complex decision-making process into several simpler decisions based on the user-defined thresholds, thus making each decision easier to visualize and interpret [68,69]. This machine learning algorithm has many advantages over the other common supervised classification algorithms, such as spectral angle mapper (SAM) or maximum likelihood classification [70]. Besides flexibility and simplicity, decision trees are adaptive to big remotely sensed datasets, such as hyperspectral images that usually carry noise and have non-linear relationships between the image attributes. Additionally, they are non-parametric during multistage decision making and therefore do not assume the distribution of input data.

In the root node of the decision tree, which is composed of the whole image data, a threshold value of 2.135 for the NI is introduced. The same NI but with lower threshold values, e.g., 2.1, 2.082, and 2.063, are introduced in the subsequent split nodes. Based on these threshold values, the image data is recursively partitioned at each node producing the leaf nodes, each representing an Nd class label assigned to each threshold value. This framework is supposed to subdivide the hyperspectral image data into different classes based on the decision logic. Each class represents a group of pixels containing a specific Nd concentration. The delineation of the spatial distribution of Nd in the samples will be useful in analyzing the REE potential of Sillai Patti carbonatite.

### 3.5. Accuracy Assessment

The results of the hyperspectral image classification were subjected to an accuracy assessment. First, using Arc GIS Pro 3.0 [71], a set of 100 random sample points was generated on the classified images. The class value of the image pixel on which a sample point randomly falls was automatically stored in a field of the attribute table of the sample point feature class. The spectral profile of each of the hundred pixels was then manually observed one by one to find out if the pixels are classified correctly. Next, the ground-truth field in the attribute table of the feature class was filled with the original class values based on the observed spectral profile and the associated NI threshold value of each pixel. Finally, a confusion matrix, also known as an error matrix, was generated based on the class values assigned to the image pixels by the decision tree classifier and the original class values manually introduced. The confusion matrix is a square array that stores the classified data in rows and the reference data or original data in columns for the number of samples (pixels in this case) used for the accuracy assessment [72].

## 4. Results

**Figure 6:**
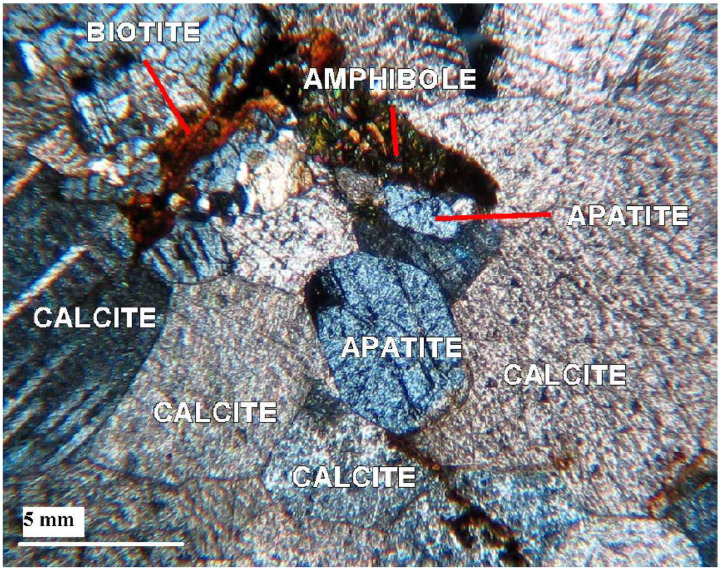
Photomicrograph of Sillai Pattai carbonatite showing the presence of primary apatite.

Petrographic results are found to be consistent with the earlier researchers, e.g., Mian [73], who divided the Sillai Patti carbonatite into biotite and amphibole sovites. In this area, both varieties of carbonatite contain 80–90% calcite, up to 6% apatite, and up to 7% iron oxide are present (Figure 6). Biotite and amphibole are present in both varieties in variable amounts. Biotite sovite has up to 7% biotite and relatively less iron oxide than amphibole sovite, which contains 50% amphibole and relatively more iron oxide. Both these varieties have trace amounts of phlogopite and pyrochlore. Amphibole sovite shows foliation as well in a few samples of ankeritic dolomite and dolomite.

**Figure 7:**
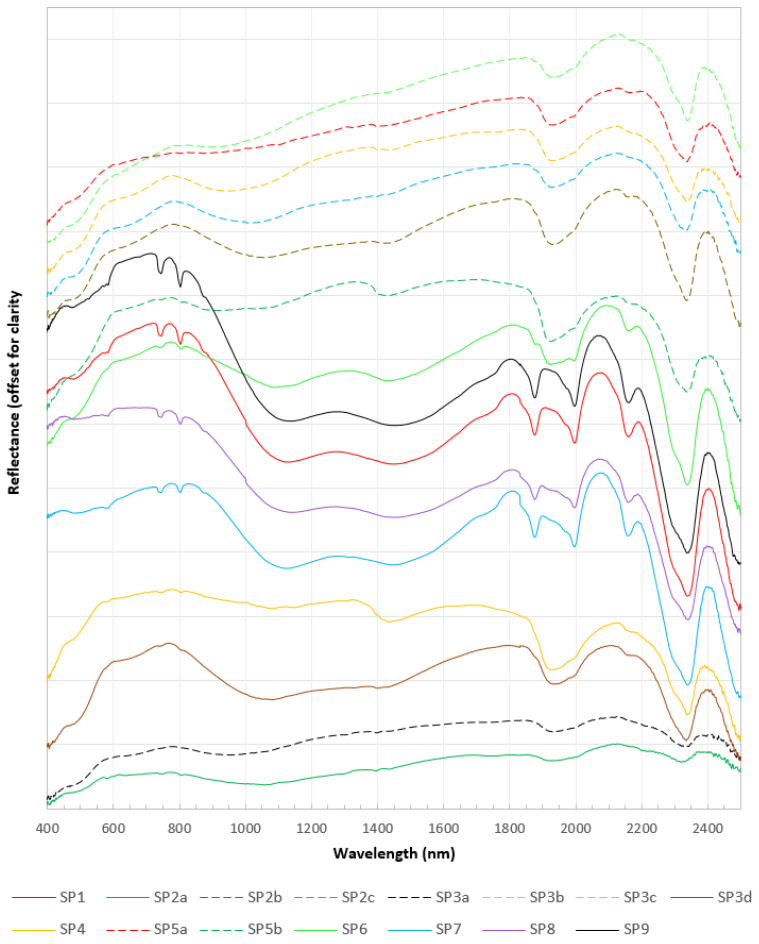
ASD spectral profiles of the samples are shown in Figure 3. Some of the samples show diagnostic absorption features of Nd at ~580, ~740, and ~810 nm. The strength of these features is highest in SP9, followed by SP1, SP8, and SP7, reflecting the highest concentration of Nd and associated REEs in the samples. Other samples have shown weaker to no Nd absorption features. The broad valley between 1000 and 1500 nm represents the iron content in the carbonatite (possibly ankerite mineral), and the diagnostic absorption features of calcite at 1875, 1995, and 2338 nm.

The reflectance spectrum of the carbonatite samples obtained through the ASD spectroradiometer effectively captured the absorption features of Nd at 745 and 810 nm (Figure 7). The strength of these features is strongest in the sample SP9 followed by the samples SP1, SP7, and SP8, which corresponds either to the higher concentration of Nd in these samples or to the coarse-grained size of the REE minerals present in the samples. In the remaining samples, these features are either very weak, e.g., in SP2a, SP3d, SP4, SP5b, and SP6, which corresponds to the lower concentration of Nd in the samples, or absent, e.g., in SP2b, SP2c, SP3a, SP3b, SP3c, and SP5a. The features may also be masked either by the featureless spectra of other minerals, e.g., magnetite, in this electromagnetic spectrum range or because of the lower albedo of a sample, e.g., in the spectrum of the SP2a collected at the darker part of the sample (Figure 4 and Figure 7). Samarium (S)-related absorption features, usually present at wavelengths 1093, 1251, and 1408 nm in the spectral profile along with Nd were not noticed in the spectrum of any of the samples.

Four of the seven carbonate absorption bands common to the spectra of all anhydrous carbonate minerals are conspicuous in the ASD spectral profile of almost all the samples. The depth of these absorption features may vary, corresponding to the varying concentrations of the carbonate minerals. The most intense and distinguishing of these four absorption features is the one centered on 2340 nm, which shows the abundance of calcite mineral in the carbonatite samples. A broad valley centered at 1200 nm, giving the whole spectrum an “M-shape“, is attributed to Fe-rich calcite, e.g., in SP1, SP2b, SP3d, SP6, SP8, SP12, and SP13 [47,74,75]. The broad absorption feature between 900 and 1000 nm in SP2c, SP3a, SP3b, and SP3c is the diagnostic feature of goethite that may have formed by the alteration of magnetite or by the exsolution from Fe-rich carbonates [67,76].

The preliminary analysis of the carbonatite samples using the spectral profiles collected through the ASD spectroradiometer was encouraging for further detailed analysis of REE distribution in the samples using hyperspectral cameras.

**Figure 8:**
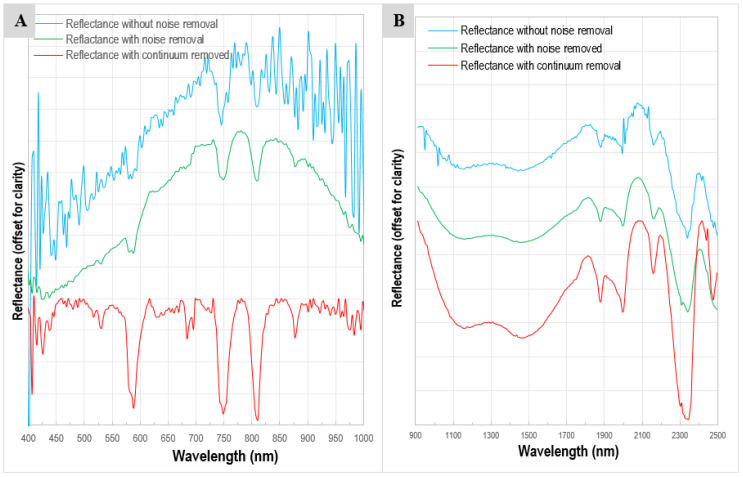
(**A**) The reflectance spectrum taken from the VNIR image of the SP7 sample. Noise-free VNIR spectra (green profile) are obtained through MNF rotation and FFT filter application applied on the noisy VNIR spectra (blue profile) that makes the noise-suppressed Nd absorption features prominent at 525, 587.5, 680, 745.91, 810.25 nm. Continuum-removed spectrum (red profile) shows the actual depth and the area of the Nd absorption feature at these absorption features. (**B**) The reflectance spectrum taken from the SWIR image of the same sample. No features of Sm, Pr, or Eu were observed in the SWIR part of the spectrum, however, the calcite absorption features are conspicuous. The valley between 1100 and 1500 nm is probably due to the Fe content.

In the VNIR image of carbonatite samples with a spectral binning of 4 (number of bands = 210), the Nd absorption features are observed in many pixels at 525, 587.5, 680, 745.91, 810.25, and 876.19 nm (Figure 8A). The absorption features may be weak and suppressed by the camera noise or masked by the magnetite spectra. Image noise removal highlighted these features, especially the absorption features centered at 745 and 810 nm, which appeared to be the most prevalent and the most prominent in almost all the Nd-rich pixels of the VNIR image. The carbonate absorption features are also observed in the SWIR images (Figure 8B).

**Figure 9:**
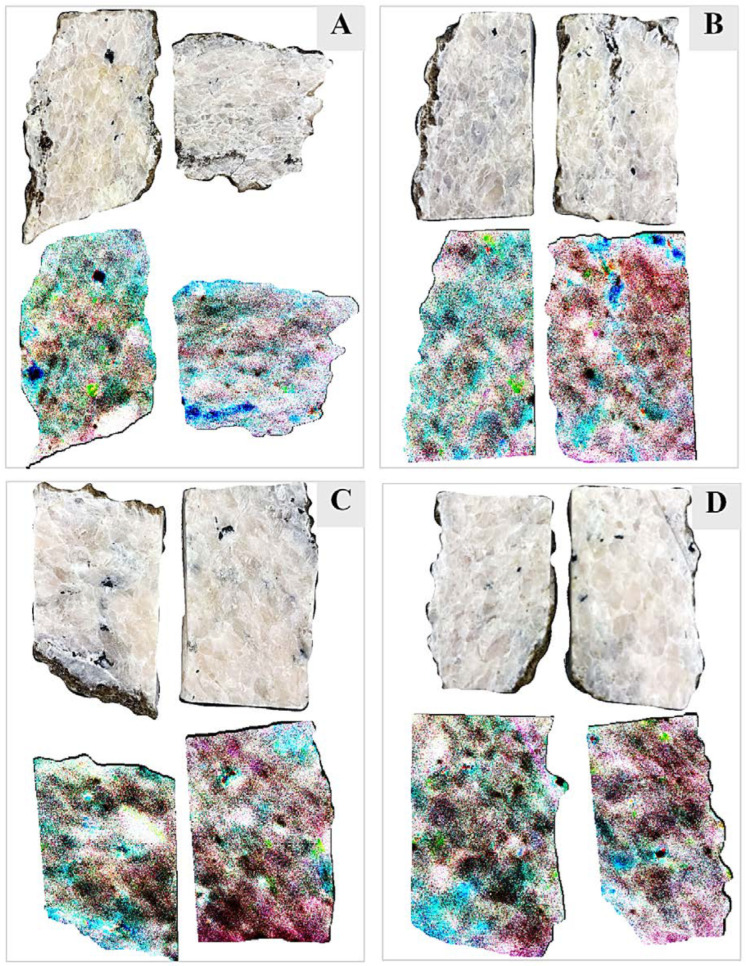
An RGB false-color composite of continuum-removed VNIR bands 69 (587.5 nm), 124 (747.91 nm), and 145 (810.25 nm). The darker the red tone of a pixel, the higher the concentration of Nd in the pixels of (**A**) sample SP1; (**B**) sample SP7; (**C**) sample SP8; (**D**) sample SP9.

To capture the Nd pixels in the VNIR image and to observe the spatial distribution of Nd across the samples, the three prominent absorption features centered at 587.5, 747.91, and 810.25 nm are used. An RGB false-color composite of continuum-removed VNIR bands 69 (587.5 nm), 124 (747.91 nm), and 145 (810.25 nm) is produced (Figure 9). All the dark brown or reddish pixels in this band combination show that the reflectance in both the green band (747.91 nm) and the blue band (810.25 nm) is very low, thus producing deep absorption bands along these wavelengths corresponding to the higher concentration of Nd. In other words, the darker the red tone of a pixel, the higher the concentration of Nd in the pixel.

**Figure 10:**
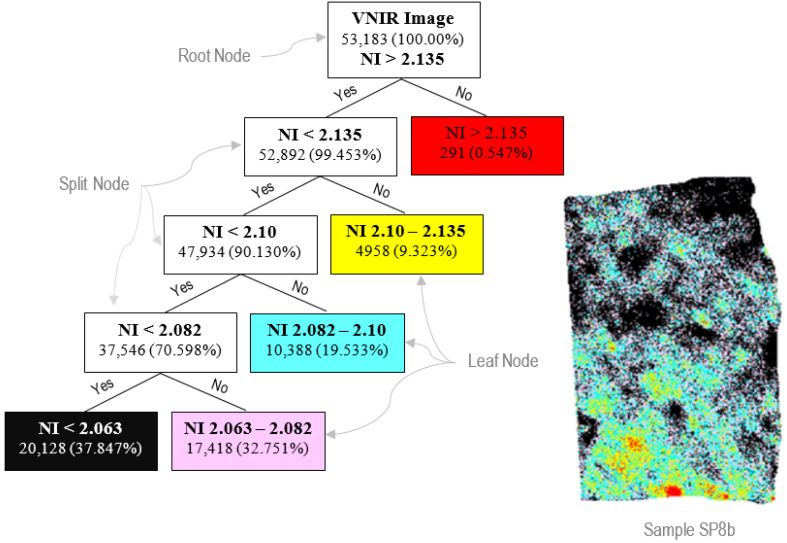
A decision tree classifier is used to delineate the quantitative spatial distribution of Nd in the sample SP8b. The root node represents the original VNIR image of the sample SP8b and calculates the total number of pixels in the image. The classifier splits the total number of pixels into different classes based on the NI value introduced at the root node as well as each of the split nodes, and calculates the percentage of pixels included in each of the five classes. The pixel distribution of each of the classes is shown in the sample image.

**Figure 11:**
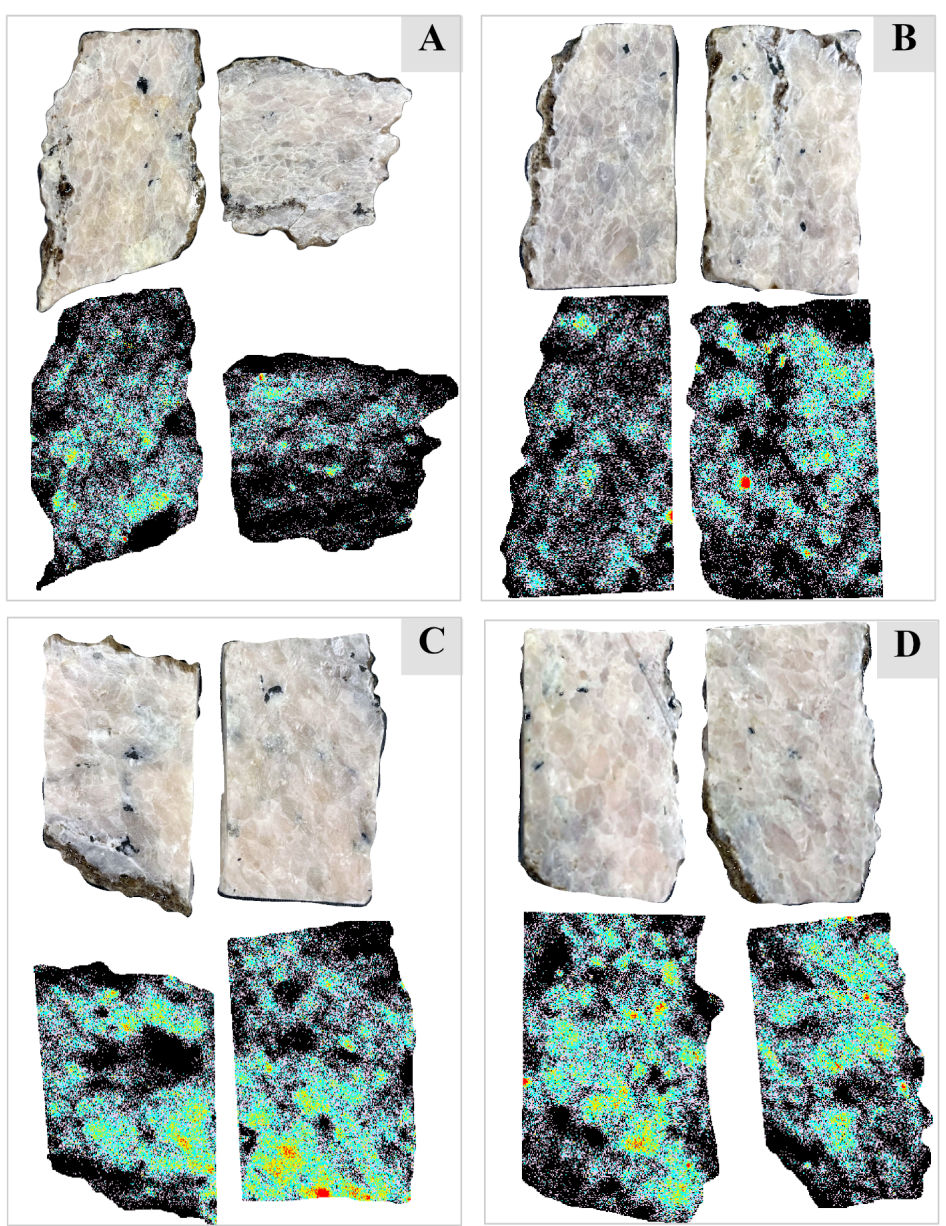
VNIR continuum-removed true-color hyperspectral images of the carbonatite samples along with the images classified using the decision tree algorithm. Based on the depth of the Nd absorption feature at ~810 nm, the relative abundance of the Nd in the samples is broken down into five classes (class 1: red pixels showing the highest Nd concentration; class 2: yellow pixels; class 3: cyan pixels; class 4: lemonade pixels; class 5: black showing little to no Nd). (**A**) sample SP1; (**B**) sample SP7; (**C**) sample SP8; (**D**) sample SP9.

Although the spatial distribution of Nd in the samples can be visualized in this RGB image, the quantification of the Nd-rich area is obtained through the decision tree classification. The classes produced by the decision tree classifier (Figure 10) using the NI defined earlier correlate well with the RGB image. The red pixels are the hot spots containing the highest Nd concentration, correlating with the pixels having the darkest tone in the RGB image (Figure 11). For each image, the decision tree classifier also calculated the number and percentage of the pixels in each class (Table 1).

## 5. Discussion

Although determining the absolute concentration of minerals and elements in a rock is not possible using hyperspectral studies, relative quantification can be undertaken using the depth and area of the diagnostic absorption features in a hyperspectral profile of the material. The NI proposed in this study, when applied to the hyperspectral images of the Sillai Patti carbonatite samples, identified the pixels containing Nd and classified the image based on the relative abundance of Nd in the samples. Using the color slices and the statistics of each slice, it was observed that the continuum-removed image has pixels with Nd absorption features as deep as 0.85 and as shallow as 0.99, with NI values ranging from as high as 2.54 and as low as 2.00, respectively.

The accuracy assessment of the classified VNIR images is shown in Table 2. The kappa coefficient of 0.817 produced from the user and producer accuracy shows that the proposed NI is useful in mapping the Nd concentration in the carbonatite samples using the decision tree classification algorithm.

The presence of Nd and associated REEs in the Sillai Patti carbonatite is confirmed by geochemical analysis conducted by Khattak [33] using the Induced Coupled Plasma-Mass Spectrometry (ICP-MS) technique. This study reported average concentrations of Nd at 314 ppm, cerium (Ce) at 915 ppm, and lanthanum (La) at 519 ppm. Although the absolute Nd concentration was not calculated in the current study, a relative abundance of Nd in the samples was evaluated with encouraging results for the detailed geochemistry of the carbonatite for Nd and other associated REEs. Identification and quantification of Nd concentration are preferred over other REEs for evaluation of a carbonatite REEs potential for two reasons: First, Nd is the most abundant LREE in carbonatites and is found in association with many other REEs in minerals, thus behaving as a major pathfinder to the associated REEs. Second, Nd has among the most prominent or the strongest unique absorption features of the REEs, mainly in VNIR and a few in the SWIR part of the spectrum [77]. In this study, Nd is preferred to other REEs and only the 810.25 nm absorption feature is used because this absorption feature does not overlap with those of other common rock-forming minerals or other REEs [21,78]. For the NI, this particular band is preferred over other absorption bands of Nd because this band has been proved as the most robust spectral proxy for Nd content, with a correlation value of r^2^ = 0.805 between the absorption area and Nd content [77].

Based on the carbonate minerals present, carbonatites are usually classed as calcite-carbonatite (sovite), dolomite-carbonatite (rauhaugite), and ankerite-carbonatite (beforsite). Seven carbonate bands are common to all anhydrous carbonate minerals [79,80,81,82]. Based on the presence of higher amounts of calcite observed in the thin section and the spectral profiles obtained both through the ASD spectroradiometer and the hyperspectral cameras, the Sillai Patti carbonatite samples are calcite-carbonatite sovite. Although dolomite or ankerite absorption features at 2316 nm [81] are absent in the spectral profiles, and thus the samples cannot be rauhaugite or beforsite, a wide valley in some spectral profiles shows the presence of Fe-rich calcite (Figure 7 and Figure 8B) [77].

Feng et al. [83] used the occurrence of apatite-hosted melt inclusions from the Ulgii Khiid carbonatites, Mongolia, to reconstruct the evolution of REE concentrations in early, primary carbonatite magma, and concluded that phosphorous (P) is known to be a key player in defining the REE budget during the carbonatite evolution. Among the three immiscible liquid phases (i.e., phosphate-, silicate-, and Fe-silicate-melt) produced during heating-quenching experiments, the initially produced phosphate melt incorporated the highest amount of REE content, causing the later stage carbonatite magmas to be depleted in REE and P content. The higher concentrations of magmatic apatite in Sillai Patti carbonatite, along with the strong spectral signatures and deep absorption features of Nd, are consistent with the findings of Feng et al. [83], and highlight the requirement for further studies on reconstructing the evolution of REE concentrations in the carbonatite.

## 6. Conclusions

This work quantified the spatial distribution of Nd and tested for the presence of associated REEs, e.g., Sm, Pr, and Eu, using spectral signatures and decision tree classification in the laboratory-based hyperspectral data of Sillai Patti carbonatite samples. A preliminary non-destructive sample examination was performed through an ASD spectroradiometer, and the Nd presence was confirmed in most of the field-collected samples based on the diagnostic absorption bands of Nd. However, none of the samples’ spectral profiles showed the absorption features of Sm, Pr, or Eu. Strong absorption features dominantly at 2340 nm in the spectral profiles suggested that the carbonatite samples are rich in calcite, which is consistent with the thin section studies of the samples as well as the previous studies that characterize Sillai Patti carbonatite as a calcite-carbonatite sovite.

Detailed pixel-based analysis of Nd spectral signatures was performed on the hyperspectral data collected in a laboratory environment using VNIR and SWIR hyperspectral cameras. It was observed that the spectral position of Nd absorption bands does not change in any of the Nd-containing pixels. This confirms the earlier work of researchers that showed the spectral signature of Nd is independent of the mineralogy containing it. However, the depth of these absorption features varies across the Nd pixels as a function of Nd concentration in the carbonatite samples. This spatial variation in Nd concentration across the samples was visualized in a false-color RGB image that is produced using the bands 69 (587.5 nm), 124 (747.91 nm), and 145 (810.25 nm) of the continuum-removed VNIR hyperspectral data. These wavelengths cover the most prominent and unmasked absorption band of Nd at ~810 nm. An NI is proposed to classify the pixels based on the depth of this absorption feature, and the decision tree classifier introduces different threshold values of the NI. The classified image, which correlates well with the RGB false color image, quantifies the Nd concentration in the carbonatite samples. Based on the minimum Nd concentration in a carbonatite sample that is detectable in the spectral signatures, it is concluded that the Sillai Patti carbonatite samples host a significant concentration of Nd, with some parts of the samples having Nd higher than 1000 ppm.

## Figures and Tables

**Table 1:** The percentage of pixels present in each class of the images.

Samples	Class 1 (NI > 2.135)	Class 2 (NI 2.135–2.1)	Class 3 (NI 2.082–2.1)	Class 4 (NI 2.063–2.082)	Class 5 (NI < 2.063)
SP1a	0.068%	2.736%	11.396%	30.961%	54.839%
SP1b	0.029%	1.028%	5.903%	22.431%	70.610%
SP7a	0.046%	1.282%	7.839%	29.042%	61.792%
SP7b	0.201%	3.021%	12.243%	29.986%	54.550%
SP8a	0.154%	7.079%	17.768%	29.669%	45.330%
SP8b	0.547%	9.323%	19.533%	32.751%	37.847%
SP9a	0.289%	6.963%	17.745%	31.776%	43.227%
SP9b	0.168%	5.708%	16.924%	32.739%	44.461%
Total Class Pixels	0.194%	4.689%	13.733%	29.968%	51.416%

**Table 2:** The confusion matrix showing the accuracy assessment of the classified images.

ClassValue	Class 1	Class 2	Class 3	Class 4	Class 5	Total	U_Accuracy	Kappa
**Class 1**	16.0	1.0	0.0	1.0	0.0	18.0	0.889	0
**Class 2**	0.0	27.0	4.0	0.0	0.0	31.0	0.871	0
**Class 3**	0.0	1.0	8.0	1.0	0.0	10.0	0.8	0
**Class 4**	0.0	0.0	0.0	8.0	1.0	9.0	0.889	0
**Class 5**	0.0	1.0	3.0	1.0	27.0	32.0	0.844	0
**Total**	16.0	30.0	15.0	11.0	28.0	100.0	0.0	0
**P_Accuracy**	1.0	0.9	0.5	0.7	1.0	0.0	0.86	0
**Kappa**	0	0	0	0	0	0	0	0.817

## Data Availability

Not applicable.
